# Normal Theory GLS Estimator for Missing Data: An Application to Item-Level Missing Data and a Comparison to Two-Stage ML

**DOI:** 10.3389/fpsyg.2017.00767

**Published:** 2017-05-22

**Authors:** Victoria Savalei, Mijke Rhemtulla

**Affiliations:** ^1^Department of Psychology, University of British ColumbiaVancouver, BC, Canada; ^2^Department of Psychology, University of California, DavisDavis, CA, USA

**Keywords:** missing data, structural equation modeling, item-level missing data, parcels, generalized least squares estimation

## Abstract

Structural equation models (SEMs) can be estimated using a variety of methods. For complete normally distributed data, two asymptotically efficient estimation methods exist: maximum likelihood (ML) and generalized least squares (GLS). With incomplete normally distributed data, an extension of ML called “full information” ML (FIML), is often the estimation method of choice. An extension of GLS to incomplete normally distributed data has never been developed or studied. In this article we define the “full information” GLS estimator for incomplete normally distributed data (FIGLS). We also identify and study an important application of the new GLS approach. In many modeling contexts, the variables in the SEM are linear composites (e.g., sums or averages) of the raw items. For instance, SEMs often use parcels (sums of raw items) as indicators of latent factors. If data are missing at the item level, but the model is at the composite level, FIML is not possible. In this situation, FIGLS may be the only asymptotically efficient estimator available. Results of a simulation study comparing the new FIGLS estimator to the best available analytic alternative, two-stage ML, with item-level missing data are presented.

## Introduction

This article proposes a new missing data estimator for incomplete normally distributed data under an ignorable missingness mechanism (Little and Rubin, 2002). The context is structural equation models (SEMs) and their special cases such as path analysis (Wright, 1921, 1934; Bollen, 1989). An ignorable missing mechanism (Little and Rubin, 2002) can be either MCAR (missing completely at random) or MAR (missing at random). Under an MCAR mechanism, missingness does not depend on any variables in the dataset. Under an MAR mechanism, missingness does not depend on any variables in the dataset that also contain missing values. State-of-the-art methods for dealing with ignorable missingness in the case of normal data include full information maximum likelihood (FIML; Arbuckle, 1996; Allison, 2003) and multiple imputation (MI; Rubin, 1987; Schafer, 1997). These approaches perform very similarly in large samples (Collins et al., 2001; Larsen, 2011; Yuan et al., 2012). In fact, when all the assumptions are met, FIML, which is the extension of the complete data maximum likelihood (ML) estimator, is asymptotically efficient. The FIML estimator is the default estimator for missing data in SEM software. Thus, one could say that the problem of missing data in the case when data are normally distributed and missingness is ignorable has been solved.

However, there exists one very common modeling context where FIML is not possible, namely when data are missing at the item level, but the model of interest is at the composite level. Variables in a path analysis model (of which regression is a special case) are often scale scores, where each scale is a linear composite (e.g., sum or average) of its corresponding items. Indicators of latent factors in an SEM are often parcels (sums of several raw items) rather than the raw items themselves (Little et al., 2013); parceling is common to reduce model size or to when the item-level measurement model is not of direct interest. One cannot fit such a model using FIML in standard software because the missingness is at the level of items whereas the model is at the level of composites. To implement FIML, one would need to specify a model using individual items as indicators instead of using parcels or composites. For example, suppose a researcher's analytic plan was to run a multiple regression model predicting negative affect from depression, stress, and big-5 personality factors, using common scales to measure all constructs. To implement FIML, the researcher would have to fit a latent variable SEM with latent variables to represent each of the 8 variables in the model, with as many reflective indicators as there are measured scale items. Thus, if each scale were composed of 10 items, fitting the item-level model would require fitting an SEM with 8 latent variables and 80 indicators. This model is substantially different than the analysis that the researcher intended, as it involves specifying, testing, and interpreting a joint factor structure for the items, which is an entirely different research question. In addition, if the measurement model for the items is not correctly specified, the structural parameter estimates will also be incorrect (Rhemtulla, 2016). Moreover, running such a model would almost certainly require a much larger sample size than the originally planned multiple regression model (MacCallum et al., 1999). If there had been no missing data, the researcher would have simply averaged the items on each scale and fit the regression or path model of interest to the data. When item-level missing data arise, the researcher must now find a statistically appropriate way to deal with it. Dealing appropriately with missing data should not necessitate testing a completely different model than the researcher intended.

In this paper, we assume that item-level data are continuous and normally distributed. While this is not, strictly speaking, a realistic assumption for the raw items, research has found that items that have 5–7 categories can be safely treated as continuous (Rhemtulla et al., 2012). Likert-type items with 5–7 categories are extremely common in psychological and behavioral research. Further, when the model is at the composite level, the misspecification of the distribution of the raw items that comprise the scale (i.e., treating them as continuous when they are ordinal) is likely to matter even less. For instance, studies of the relative performance of categorical vs. normal multiple imputation find, in the context of item-level missing data, that imputation under the normal model does as well as or better than imputation under the multinomial model, even when items are binary or have three categories (Wu et al., 2015). Thus, we do not consider the continuous treatment of items when interest is in the composite-level model to be a very strict assumption in practice.

We now summarize existing approaches to dealing with continuous item-level missing data when the model is at the composite level. One *ad-hoc* approach is to set the composite score to missing whenever any of the corresponding components (raw items) are missing, and then run FIML on the resulting dataset. This approach has been referred to as “scale-level” FIML. This method throws away a lot of information, and at the limit the researcher may be left with no data if all participants left at least one item unanswered on each composite (Gottschall et al., 2012).

Another *ad-hoc* approach is to create a composite score by averaging all the available items on that composite for a given participant. This approach has been referred to as “available-case” ML (ACML; Savalei and Rhemtulla, 2017) or “proration” (Mazza et al., 2015). This solution is equivalent to imputing the participant's scale mean for the missing items (Schafer and Graham, 2002). The resulting composite scores typically do not have any missing values, and the model is estimated on the composite dataset using ML. If there is some missing data remaining (e.g., some participants left all items blank), the model is estimated on the composite dataset using FIML. ACML is often the method that researchers resort to when they encounter item-level missing data, because it seems to extract the most information out of the raw data. While this method will often work fine, it does not guarantee consistency, i.e., the property that sample estimates will approach population parameter values as the sample size grows large. For example, if raw items with missing values have substantially different means and variances than items with no missing values, consistency will not hold (Schafer and Graham, 2002; Graham, 2003). In fact, neither scale-level FIML nor available-case ML have the property of consistency under a general ignorable missing data mechanism, and for this reason we do not discuss them further. Finally, a reviewer pointed out that a hybrid method is often used in practice, whereby researchers impute the person-level mean for the missing items if there are not too many of them, but if the number of missing items per person is too great, the entire composite is declared as missing. Obviously, this approach suffers from the same shortcomings.

Several theoretically justified alternatives are available to researchers instead. These alternatives produce consistent estimates under an ignorable missing data mechanism. The first option is item-level multiple imputation (item-level MI). Multiple imputation allows the user to treat missing data during the imputation stage. Missing item scores are imputed under the normal model. In the analysis stage, composite scores are created within each imputed dataset, and the model is fit to these composite scores using ML. The results are averaged across imputations using standard formulae (Rubin, 1987). The second option is two-stage ML (TSML), recently proposed by Savalei and Rhemtulla (2017). This approach represents the best currently available analytic solution to the problem of item-level missing data. It performs as well as or better than item-level MI. In fact, for a large number of imputations, this approach can be thought of as the analytic equivalent of item-level MI. The technical details behind TSML will be summarized shortly.

The third option is to run scale-level FIML while incorporating a subset of the raw items (as many as possible) into the model as auxiliary variables (Mazza et al., 2015). This approach represents a partial solution because all of the raw items cannot be used as auxiliary variables while the composite score is also in the model. Thus, while the developers of this approach refer to it as “FIML,” consistency and asymptotic efficiency are approximated but not guaranteed. This approach can also be rather unwieldy and difficult to implement. We will not discuss it further.

Thus, TSML and item-level MI are theoretically most appropriate for item-level missing data, and are equivalent in large samples and with a large number of imputations. However, TSML is not asymptotically efficient with normally distributed item-level missing data, although its efficiency is fairly high. In order to consider the problem of item-level missing data “solved,” we are seeking a solution that produces an estimator that is asymptotically efficient with normally distributed data. We now define one such solution.

We draw upon the analogy with complete data, where two asymptotically efficient estimation methods are available for SEM analysis when the data are normally distributed: ML and GLS (generalized least squares; Browne, 1974; Bollen, 1989). The main difference between these two normal-theory estimators is that GLS uses sample covariance matrix instead of the model-implied covariance matrix in the weight matrix. These methods are asymptotically equivalent when the correct model is fit to data (Shapiro, 1985; Yuan and Chan, 2005). In practice, the GLS estimator is almost never used with complete data, because research has shown that it is outperformed by ML (Ding et al., 1995; Hu and Bentler, 1998; Olsson et al., 1999, 2000). However, this does not mean that this method cannot be useful in a context when ML is not available. There is also no reason to expect that its performance with incomplete data will be the same as its performance with complete data.

In this article we extend the normal-theory GLS estimator used in SEM analysis to incomplete data, dubbing it FIGLS (“full information” GLS). This extension results in a consistent and asymptotically efficient estimator for incomplete normally distributed data under an ignorable missing data mechanism. For models that are based on the raw items, and when the model is correct, the FIGLS estimator is asymptotically equivalent to FIML. However, unlike FIML, which cannot be used to treat item-level missing data when the model is based on the composites, the FIGLS estimator is straight-forwardly adapted to this situation. It is also asymptotically efficient with such data, thus possessing theoretical advantages over TSML and item-level MI.

The rest of this article is organized as follows. First, we review the GLS estimator with complete data. Next, we present the technical details of its extension, FIGLS, to incomplete data. We then describe the extension of FIGLS to item-level missing data. For completeness, we also present the technical details of the comparison estimator for item-level missing data, TSML (Savalei and Rhemtulla, 2017). Next, we summarize the results of a simulation study comparing the FIGLS and TSML in the context of an SEM model with parcels, where missingness is at the item level. The simulation study varies sample size, percent missing data, type of missing data mechanism, and strength of inter-item correlations. We end with a discussion.

## Technical details

### ML and GLS estimators with complete normally distributed data

Let the model representation be μ = μ(θ), Σ = Σ(θ), where μ is the *p*×1 vector of population means for *p* variables, Σ is the *p*×*p* population covariance matrix, and θ is the *q*×1 vector of SEM parameters (e.g., factor loadings). Let the corresponding sample vector of means and sample covariance matrix be $\bar{x}$ and *S*. The ML fit function is given by:

(1) $$\begin{array}{r} F_{ML}=tr\left\{S\Sigma^{-1}\right\}+\log|\Sigma |-\log|S|+{\left(\bar{x}-\mu \right)}^{\prime }\Sigma^{-1}\left(\bar{x}-\mu \right), \end{array}$$

where μ = μ(θ) and Σ = Σ(θ) are structured according to the model, but this dependence on θ has been suppressed for clarity.

An important related fit function is the RLS (reweighted least squares) fit function:

(2) $$\begin{array}{r} F_{RLS}={\left(s-\sigma \right)}^{\prime }W_{RLS,cov}\left(s-\sigma \right)+{\left(\bar{x}-\mu \right)}^{\prime }\Sigma^{-1}\left(\bar{x}-\mu \right), \end{array}$$

where $W_{RLS,cov}=0.5D_{p}^{\prime }\left(\Sigma^{-1}⊗\Sigma^{-1}\right)D_{p}$, *s* = *vechS* and σ = *vechΣ* (i.e., non-redundant elements of the corresponding matrices, vectorized), and *D*_*p*_ is the 0–1 duplication matrix of order *p* (see Magnus and Neudecker, 1999, for exact definitions of the duplication matrix and the *vech* operator). The ML estimator can be equivalently viewed as the minimizer of *F*_*ML*_ or of *F*_*RLS*_, where the weight matrix in the latter fit function is iteratively updated (Lee and Jennrich, 1979). Viewing the ML estimator as the minimizer of *F*_*RLS*_ reveals the parallel between the ML and the GLS estimators.

The GLS fit function is given by:

(3) $$\begin{array}{r} F_{GLS}={\left(s-\sigma \right)}^{\prime }W_{GLS,cov}\left(s-\sigma \right)+{\left(\bar{x}-\mu \right)}^{\prime }{\text{S}}^{-1}\left(\bar{x}-\mu \right) \end{array}$$

where $W_{GLS,cov}=0.5D_{p}^{\prime }\left(S^{-1}⊗S^{-1}\right)D_{p}$. Thus, while both the ML and the GLS estimators assume normality, the ML estimator uses the model-implied covariance matrix in the weight matrix of the fit function (in reality, these are iteratively updated estimates, as the true values are not known), while the GLS estimator uses the sample covariance matrix and means in the weight matrix (Bollen, 1989).

It is important to note, in order for the extension to incomplete data to be clear, that *W*_*GLS, cov*_ is an estimate of the inverse of the asymptotic covariance matrix of *s*, and S^−1^ is an estimate of the inverse of the asymptotic covariance matrix of $\bar{x}$. With complete data, $\bar{x}$ and *S* are independent, and the fit function in (3) is split into two quadratic forms. Its alternative expression is given by:

(4) $$\begin{array}{r} F_{GLS}={\left(\begin{array}{c} s\;-\;\sigma \\ \bar{x}\;-\;\mu \end{array}\right)}^{\prime }\left(\begin{array}{cc} W_{GLS,cov} & 0\\ 0 & {\text{S}}^{-1} \end{array}\right)\left(\begin{array}{c} s\;-\;\sigma \\ \bar{x}\;-\;\mu \end{array}\right) \end{array}$$

The block-diagonal matrix in the middle is the full weight matrix *W*_*GLS*_. This is the weight matrix we will generalize to incomplete data.

### Full-information GLS estimator for incomplete normally distributed data (FIGLS)

The mean and the covariance structure must be estimated jointly with incomplete data, as they are no longer independent. For this reason, it is helpful to rewrite the model representation as β = β(θ), where β = (σ′, μ′)′, a [0.5*p*(*p* + 1) + *p*] × 1 vector.

The FIML estimator (Arbuckle, 1996; Allison, 2003) is the extension of the ML estimator to incomplete data. Unfortunately, it is no longer straight-forward to define this estimator as the minimizer of a fit function analogous to *F*_*ML*_. Further, summary statistics analogous to $\bar{x}$ and *S* are not directly available with incomplete data, and instead the estimate of θ is obtained directly from the raw data by maximizing the incomplete data log-likelihood under the proposed model. We omit the details as this method is not directly relevant to the developments in this paper.

However, an important special case of the FIML approach is necessary for the development of the GLS estimator for incomplete data. If the incomplete data log-likelihood is maximized under the saturated model (i.e., no structure is imposed on μ and Σ), the resulting “saturated” FIML estimates $\hat{\mu }$ and $\hat{\Sigma }$ are the incomplete data analogs of $\bar{x}$ and *S*. These have sometimes been referred to as the “EM means” and the “EM” covariance matrix (e.g., Enders and Peugh, 2004), because they are most straight-forwardly obtained via the application of the EM algorithm (e.g., the *norm* package in R).

Because with incomplete data, $\hat{\mu }$ and $\hat{\Sigma }$ are no longer independent, their joint asymptotic covariance matrix is needed. Let the vector of saturated FIML estimates be $\hat{\beta }={\left({\hat{\sigma }}^{\prime },{\hat{\mu }}^{\prime }\right)}^{\prime }$. Yuan and Bentler (2000) gave an explicit expression for the estimate of the inverse of the asymptotic covariance matrix of $\hat{\beta }$ under MCAR, and Savalei (2010) gave the corresponding expression under MAR. The exact expressions are omitted here. We denote the estimate of the asymptotic covariance matrix of $\hat{\beta }$ by ${\hat{\Omega }}_{\beta }$. The weight matrix for the new GLS estimator with missing data is then set to $W_{FIGLS}={\hat{\Omega }}_{\beta }^{-1}$. The FIGLS fit function for incomplete normally distributed data is given by:

(5) $$\begin{array}{r} F_{FIGLS}={\left(\hat{\beta }-\beta \right)}^{\prime }W_{FIGLS}\left(\hat{\beta }-\beta \right) \end{array}$$

This function parallels the expression in (4) for complete data. As with complete data, the FIGLS and the FIML estimators are asymptotically equivalent when the distributional assumptions are met and the model is true. They are both asymptotically efficient. However, the FIML estimator works quite well with incomplete data, and thus there may not be much application for the FIGLS estimator in the straight-forward situation where the model is based on the raw items that contain missing data, although its study and performance relative to FIML is certainly encouraged.

### The application of FIGLS when the model is at the composite level

We now define the extension of FIGLS to the situation when data are missing at the item level while the model is at the composite level. The FIML estimator is no longer possible without specifying and fitting a model to the raw items. In contrast, the FIGLS estimator is still available because the weight matrix for the composite model is a straightforward function of the item-level means and covariance matrix under a saturated structure. FIGLS is also asymptotically efficient.

In this setup, the researcher is now interested in grouping the *p* variables into *k* composites. It is not a requirement that all composites have an equal number of items, and it may be that some composites consist of a single item. Let *C* be the *k*×*p* matrix of 0′s and 1′s that corresponds to the linear transformation of the *p* components into the *k* composites. The structural equation model of interest to the researcher is at the composite level: μ_*C*_ = μ_*C*_(θ), Σ_*C*_ = Σ_*C*_(θ), where μ_*C*_ = *Cμ* and $\Sigma_{C}=C\Sigma {\text{C}}^{\prime }$ are the population means and covariances of the composites. The parameter θ is now assumed to structure the means and the covariances of the composite scores, not of the original raw scores. As before, it is helpful to rewrite the model in vectorized form as β_*C*_ = β_*C*_(θ), where $\beta_{C}={\left({\sigma^{\prime }}_{C},\mu_{C}^{\prime }\right)}^{\prime }$, a [0.5*k* (*k* + 1) + *k*] × 1 vector.

The corresponding saturated model estimates of the means and covariances of the composites can be obtained from the corresponding saturated model estimates for the raw items by ${\hat{\mu }}_{C}\;=\;C\hat{\mu }$ and ${\hat{\Sigma }}_{C}=C\hat{\Sigma }{\text{C}}^{\prime }$. We arrange these estimates in a vector ${\hat{\beta }}_{C}={\left({\hat{\sigma }}_{C}^{\prime },{\hat{\mu }}_{C}^{\prime }\right)}^{\prime }$. It is convenient to relate the saturated estimates for the composites and the raw items, as follows: ${\hat{\beta }}_{C}=C_{\beta }\hat{\beta }$, where $C_{\beta }=\left[\begin{array}{cc} D_{k}^{+}\left(C⊗C\right)D_{p} & 0\\ 0 & C \end{array}\right]$, a [0.5*k* (*k* + 1) + *k*] × [0.5*p*(*p* + 1) + *p*] matrix, and $D_{k}^{+}$ is the Moore-Penrose inverse of the duplication matrix of order *k* (Magnus and Neudecker, 1999). It follows that the asymptotic covariance matrix of ${\hat{\beta }}_{C}$ is related to the asymptotic covariance matrix of $\hat{\beta }$ as ${\hat{\Omega }}_{\beta_{C}}={C_{\beta }{\hat{\Omega }}_{\beta }C}_{\beta }$.

The weight matrix for the FIGLS estimator with composites is given by $W_{FIGLS,C}={\hat{\Omega }}_{\beta_{C}}^{-1}$, and the FIGLS fit function adapted to composites is given by:

(6) $$\begin{array}{r} F_{FIGLS,C}={\left({\hat{\beta }}_{C}-\beta_{C}\right)}^{\prime }W_{FIGLS,C}\left({\hat{\beta }}_{C}-\beta_{C}\right) \end{array}$$

Because the weight matrix is optimal in the sense that it is the inverse of the asymptotic covariance matrix of ${\hat{\beta }}_{C}$ (Browne, 1974; Shapiro, 1985), if optimization is done with this weight matrix in any software that allows for a custom weight matrix (such as *lavaan*; Rosseel, 2012), the default printed standard errors and tests statistic will be valid, and the parameter estimates will be asymptotically efficient. For completeness, we provide the equations for the standard errors and the test statistic here.

The asymptotic covariance matrix of the FIGLS parameter estimates $\hat{\theta }$ obtained by minimizing (6) is given by ${\overset{ˇ}{\Omega }}_{\theta }={\left(\overset{ˇ}{\Delta }{\hat{\prime \Omega }}_{\beta_{C}}^{-1}\overset{ˇ}{\Delta }\right)}^{-1}$, where $\overset{ˇ}{\Delta }={\frac{\partial \beta_{C}\left(\theta \right)}{\partial \theta^{\prime }}|}_{\theta =\overset{ˇ}{\theta }}$, the matrix of model derivatives. Standard errors are obtained from the diagonal elements of ${\overset{ˇ}{\Omega }}_{\theta }$. The model test statistic is given by ${T_{FIGLS,C}=\left(N-1\right)F}_{FIGLS,C}\left(\overset{ˇ}{\theta }\right)$, where *N* is sample size. When the model is correct, this statistic has an asymptotic chi-square distribution with [0.5*k* (*k* + 1) + *k*] − *q* degrees of freedom.

### The two-stage estimator when the model is at the composite level (TSML)

We now summarize the details of the TSML estimator (Savalei and Rhemtulla, 2017), which will be used for comparison in the simulation study. This approach is not asymptotically efficient, unlike the FIGLS estimator. Its efficiency is high, however, and because of the greater simplicity of its fit function it may be preferred in smaller samples. The TSML fit function is:

(7) $$\begin{array}{rcl} F_{TSML} & = & tr\left\{{\hat{\Sigma }}_{C}\Sigma^{-1}\right\}+\log|\Sigma |-\log|{\hat{\Sigma }}_{C}|\\ +\;{\left({\hat{\mu }}_{C}\;-\;\mu \right)}^{\prime }\Sigma^{-1}\left({\hat{\mu }}_{C}\;-\;\mu \right) \end{array}$$

This fit function is essentially the complete data ML fit function in (1), but with the composite saturated estimates ${\hat{\mu }}_{C}$ and ${\hat{\Sigma }}_{C}$ replacing $\bar{x}$ and *S*. As with FIGLS, the composite saturated estimates are obtained using the equations ${\hat{\mu }}_{C}=C\hat{\mu }$ and ${\hat{\Sigma }}_{C}=C\hat{\Sigma }{\text{C}}^{\prime }$, where $\hat{\mu }$ and $\hat{\Sigma }$ are the saturated estimates for the raw items (e.g., obtained via the *norm* package in R). In words, the TSML parameter estimates $\tilde{\theta }$ are obtained by “forgetting” there was ever missing data. This method has intuitive appeal, but if one simply plugs in the saturated estimates ${\hat{\mu }}_{C}$ and ${\hat{\Sigma }}_{C}$ into standard SEM software and fits the model, the default standard errors and test statistic will be incorrect. This method is not asymptotically efficient and requires adjustments to standard errors and test statistic (Savalei, 2014). These corrections require special programming as they are currently not automated in software; however, they will soon be available in *lavaan* (Rosseel, 2012). For completeness, we give the exact equations here.

Let the model-implied means and covariances constructed from TSML estimates $\tilde{\theta }$ be ${\tilde{\mu }}_{C}$ and ${\tilde{\Sigma }}_{C}$, and their vectorized version ${\tilde{\beta }}_{C}={\left({\left(vech{\tilde{\Sigma }}_{C}\right)}^{\prime },{\tilde{\mu }}_{C}^{\prime }\right)}^{\prime }$. The correct asymptotic covariance matrix of $\tilde{\theta }$ is given by ${\tilde{\Omega }}_{\theta }={\left({\tilde{\Delta }}^{\prime }\tilde{H}\tilde{\Delta }\right)}^{-1}{\tilde{\Delta }}^{\prime }\tilde{H}{\hat{\Omega }}_{{ß}_{C}}\tilde{H}{\tilde{\Delta }\left({\tilde{\Delta }}^{\prime }\tilde{H}\tilde{\Delta }\right)}^{-1}$, where $\tilde{\Delta }$ is the matrix of model derivatives evaluated at $\tilde{\theta }$, and $\tilde{H}=\left[\begin{array}{cc} 0.5D_{k}^{\prime }\left({\tilde{\Sigma }}_{C}^{-1}⊗{\tilde{\Sigma }}_{C}^{-1}\right)D_{k} & 0\\ 0 & {\tilde{\Sigma }}_{C}^{-1} \end{array}\right]$, the complete-data normal-theory weight matrix evaluated at $\tilde{\theta }$. Note that $\tilde{H}$ has the same form as the RLS (and therefore, asymptotically, ML) weight matrix in equation (2) and the GLS matrix in equations (3) and (4). Standard errors for $\tilde{\theta }$ are obtained from the diagonal elements of ${\tilde{\Omega }}_{\theta }$. A good model test statistic is the normal-theory residual-based statistic $T_{TSML}=\left(N-1\right){\left({\hat{\beta }}_{C}-{\tilde{\beta }}_{C}\right)}^{\prime }\left({\hat{\Omega }}_{\beta_{C}}^{-1}-{\hat{\Omega }}_{\beta_{C}}^{-1}\tilde{\Delta }{\left({\tilde{\Delta }}^{\prime }{\hat{\Omega }}_{\beta_{C}}^{-1}\tilde{\Delta }\right)}^{-1}{\tilde{\Delta }}^{\prime }{\hat{\Omega }}_{\beta_{C}}^{-1}\right)\left({\hat{\beta }}_{C}-{\tilde{\beta }}_{C}\right)$ (Savalei and Bentler, 2009). This statistic has an asymptotic chi-square distribution with [0.5*k* (*k* + 1) + 1] − *q* degrees of freedom.

## Methods

We conducted a simulation study to provide a first evaluation of the new FIGLS estimator when the model is based on composites. The design of this study parallels that of Savalei and Rhemtulla (2017). Because the best-performing analytic method in that study was TSML, we include it as a comparison method.

### Data generation

The number of raw items in the simulated data was set to ***p*** = **27**, and the number of composites created from these raw items was set to ***k*** = **9**. In this study, all composites consisted of 3 items. The raw items were set to follow a hierarchical factor model with 9 first-order and 3 second-order factors. Each first order factor had three indicators, and each second order factor had three first-order factors as indicators. Two models were used. In Model 1, the values of first-order factor loadings for each first-order factor were 0.3, 0.4, and 0.5 (averaging to 0.4); in Model 2, they were 0.6, 0.7, and 0.8 (averaging to 0.7). Values of the second-order factor loadings were set to 0.6, 0.7, and 0.8 for each second-order factor (averaging to 0.7) in both models. Second-order Factor 2 was regressed on second-order Factor 1 with β = **0.6;** second-order Factor 3 was regressed on second-order Factor 2 with β = **0.6**. The variances of all observed and latent variables were 1.

**Figure 1:**
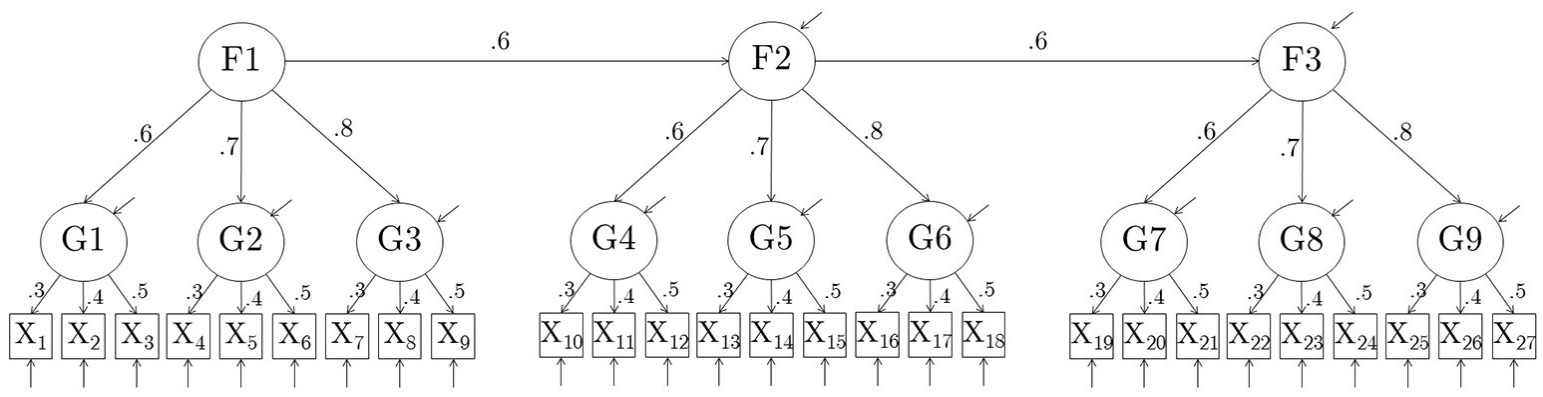
**Model 1 used to generate complete data**. In model 2, first-order factor loadings were {0.6, 0.7, 0.8} instead. Variances of all observed and latent variables are 1.

**Figure 2:**
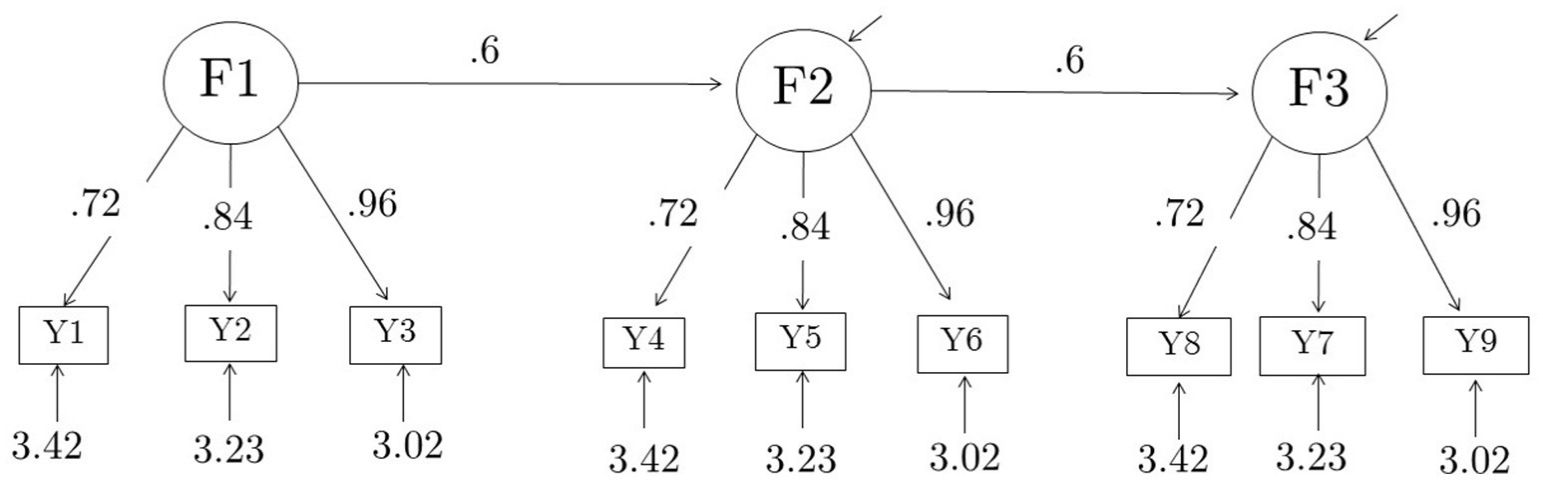
**Composite model, shown with true parameter values for Model 1**. Standardized factor loadings for Model 1 are {0.363, 0.423, 0.484} for each factor. The corresponding true parameter values for Model 2 are given in Table 1. Standardized factor loading values for Model 2 are {0.52, 0.60, 0.69}. These values were derived algebraically from the corresponding values for the components; the derivations were verified empirically by fitting the analysis model to the population covariance matrices of the composites. The analysis model was fit with (residual) factor variances fixed to their true values, and all loadings, latent regression coefficients, and indicator residual variances freely estimated.

Nine composites were formed out of the 27 raw items by adding up the 3 indicators of each of the 9 first-order factors, thus creating parcels consisting of indicators of first-order factors. The correct model for the composites, which can be derived from the model for the raw items, is a 3-factor model with three indicators per factor, with standardized loadings of each factor equal to 0.34, 0.40, 0.46 for Model 1, and 0.46, 0.54, and 0.62 for Model 2. Unstandardized parameter values for the composite models are summarized in Table 1. The raw model for the 27 items and the parceled model for the 9 items are shown in Figures 1, 2, respectively.

**Table 1:** **True parameter values for the composite model**.

	**Model 1**	**Model 2**
Unstandardized factor loadings (for each factor)	0.72, 0.84, 0.96	1.26, 1.47, 1.68
Standardized factor loadings (for each factor)	0.36, 0.42, 0.48	0.52, 0.60, 0.69
Residual variances of indicators (for each factor)	3.42, 3.23, 3.02	4.33, 3.76, 3.10
Factor regression coefficients (F1->F2, F2->F3)	0.6, 0.6	0.6, 0.6
Factor residual variances (F2, F3)	0.64, 0.64	0.64, 0.64

Complete data on the raw items were generated in R by drawing samples from a multivariate normal distribution. Sample sizes were set to *N* = 200, 400, or 600. One thousand datasets were drawn in each condition. Next, nine incomplete datasets were created from each complete dataset. These corresponded to the intersection of the three “percent missing data” conditions and the three “missingness mechanism” conditions, described next.

Percent missing data was set to be 5, 15, or 30% for 15 out of the 27 raw items. The remaining items had complete data. The 15 items with missing data were partitioned into 6 sets: {*X*_1_, *X*_5_, *X*_9_}, {*X*_10_, *X*_11_}, {*X*_14_, *X*_15_, *X*_16_, *X*_18_}, {*X*_20_, *X*_21_}, {*X*_22_, *X*_24_}, and {*X*_25_, *X*_26_}. Items within each set were missing jointly, while missingness across sets was generated independently.

The missing data mechanisms were MCAR, MAR-linear, and MAR-nonlinear. MCAR missingness was generated by randomly picking a row of the dataset and creating missing data for the items within a set, and repeating until the desired percent of missing data per item was reached, for each of the six sets. MAR missingness was generated by using a set of six complete items {*X*_2_, *X*_12_, *X*_13_, *X*_19_, *X*_23_, *X*_27_} as conditioning variables for each of the six sets of incomplete items: For a randomly picked row, the corresponding set was deleted if the corresponding conditioning variable was >0 (MAR-linear) or >0.67 in absolute value (MAR-nonlinear), repeating until the desired percent of missing data per item was reached.

### Implementation of methods

Both FIGLS and TSML implementation require the saturated FIML estimates $\hat{\mu }$ and $\hat{\Sigma }$ (arranged in a vector $\hat{\beta }$). These were obtained by running the saturated model on the full 27-item incomplete datasets using *lavaan* 0.5–18 (Rosseel, 2012). The associated asymptotic covariance matrix ${\hat{\Omega }}_{\beta }$ was also obtained from *lavaan*. The corresponding saturated estimates of the composites, ${\hat{\mu }}_{C}$ and ${\hat{\Sigma }}_{C}$ (arranged in a vector ${\hat{\beta }}_{C}$) and their associated asymptotic covariance matrix, ${\hat{\Omega }}_{\beta_{C}}$, were computed using the equations relating these quantities (see Section Technical Details).

To obtain TSML estimates, the correct model for composites was fit to data using the complete data ML estimation in *lavaan*, with ${\hat{\mu }}_{C}$ and ${\hat{\Sigma }}_{C}$ supplied as “sample” means and covariance matrix. The correct standard errors and the normal-theory residual-based statistic were computed in R using the equations in Section Technical Details. The matrix of model derivatives $\tilde{\Delta }$ necessary for these computations was obtained from *lavaan*.

To obtain FIGLS estimates, the correct model for composites was fit using WLS estimation in *lavaan*, with the weight matrix specified to be ${\hat{\Omega }}_{\beta_{\text{C}}}^{-1}$ (lavaan allows for a custom weight matrix specification, using the “wls.v” option). Default *lavaan* computations of standard errors and the model chi-square were used, as these are already correct for asymptotically efficient estimators.

For both methods, the model for the composites was fit with the variance of Factor 1 and the residual variances of Factors 2 and 3 fixed to their true values for identification. All loadings, latent regression coefficients, and indicator residual variances were freely estimated. Sample syntax for both TSML and FIGLS is provided on the Open Science Framework.

### Outcome variables

The following dependent variable measures were used to compare the two methods:

- Number of useable replications (after removing convergence failures, condition codes, and outliers)
- Bias of parameter estimates
- Empirical standard deviations of parameter estimates (a measure of efficiency)
- Root mean square error of parameter estimates (a joint measure of bias and efficiency)
- Coverage of 95% confidence intervals (a combined measure of bias and quality of standard error estimates)
- Type I error rates of the model test statistic.

An outlier was defined as a parameter estimate of either a factor loading or a latent regression coefficient that exceeded 10 in absolute value. Including replications where estimates were this far from their true values (see Table 1) would bias the summary statistics across replications.

Bias was computed as the average deviation of each parameter estimate from its true value across replications. Empirical standard deviations were computed as the square-root of the average squared difference between the parameter estimate and its average in that cell. Coverage was computed as the number of times out of the number of useable replications that a 95% confidence interval (CI) contained the true value of the parameter. To simplify presentation, all these measures were further averaged across parameter estimates of each type. Results for residual variances and for means were not examined as these parameters are rarely relevant in confirmatory factor analysis. Thus, the results were examined jointly for the 9 factor loadings and for the 2 regression coefficients. Finally, Type I error rates were computed as the number of times out of the number of useable replications that a test statistic produced a *p* < 0.05.

## Results

### Number of usable replications

Convergence failures, condition codes, and outliers were generally limited to Model 1, and were highest in number with *N* = 200. The number of problematic replications outside of this intersection of conditions was negligible. For Model 1 with *N* = 200, the GLS estimator produced greater convergence rates, but more condition codes and outliers. As a result, the total number of usable replications in these conditions was very similar for the two methods. Table 2 gives the number of usable replications in all study conditions. It is clear that one cannot recommend one method over another based on the number of useable replications. The remaining results are based on these replications only.

**Table 2:** **Number of useable replications across study conditions**.

***N***	**Missing**	**%**	**Model1**		**Model2**	
	**mech**	**Miss**	**FIGLS**	**TSML**	**FIGLS**	**TSML**
200	MCAR	5	942	943	1,000	1,000
		15	921	916	1,000	1,000
		30	873	874	1,000	1,000
	MAR.lin	5	949	947	1,000	1,000
		15	926	927	1,000	1,000
		30	851	858	1,000	1,000
	MAR.nl	5	939	948	1,000	1,000
		15	920	931	1,000	1,000
		30	851	861	1,000	1,000
400	MCAR	5	998	999	1,000	1,000
		15	998	999	1,000	1,000
		30	992	988	1,000	1,000
	MAR.lin	5	999	1,000	1,000	1,000
		15	997	997	1,000	1,000
		30	989	990	1,000	1,000
	MAR.nl	5	998	998	1,000	1,000
		15	994	996	1,000	1,000
		30	992	991	1,000	1,000
600	MCAR	5	1,000	1,000	1,000	1,000
		15	1,000	1,000	1,000	1,000
		30	999	999	1,000	1,000
	MAR.lin	5	1,000	1,000	1,000	1,000
		15	1,000	1,000	1,000	1,000
		30	998	998	1,000	1,000
	MAR.nl	5	1,000	1,000	1,000	1,000
		15	1,000	1,000	1,000	1,000
		30	999	999	1,000	1,000

“*MAR.lin” and “MAR.nl” stand for MAR-linear and MAR-nonlinear missing mechanisms, respectively*.

### Bias of parameter estimates

Average bias in the loadings and the latent regression coefficients was generally small for both methods and across all study conditions. As expected, bias decreased with sample size. The results are shown in Table 3. These results correspond to raw bias, and should be interpreted as the average deviations from the true parameter values. As Table 1 shows, the unstandardized values of the factor loadings were quite large. From this perspective, the largest observed value of the average bias for the factor loadings, which was 0.01, is tiny. On the other hand, the latent regression coefficients were 0.6 in the population, whereas the largest observed value of the average bias was 0.132 for FIGLS and 0.112 for TSML (both under Model 1). However, these values improved quite quickly with increasing sample size, even in the worst missing data conditions.

**Table 3:** **Average bias in factor loadings and latent regression coefficients across all study conditions**.

***N***	**Missing**	**%**	**Factor loadings**				**Latent regression coefficients**			
	**mech**	**Miss**	**Model 1**		**Model 2**		**Model 1**		**Model 2**	
			**FIGLS**	**TSML**	**FIGLS**	**TSML**	**FIGLS**	**TSML**	**FIGLS**	**TSML**
200	MCAR	5	−0.006	−0.004	−0.037	−0.015	0.093	0.071	0.035	0.017
		15	−0.003	0.001	−0.038	−0.016	0.114	0.085	0.038	0.019
		30	0.004	0.009	−0.041	−0.020	0.132	0.104	0.046	0.025
	MAR.lin	5	−0.010	−0.007	−0.037	−0.015	0.105	0.082	0.034	0.017
		15	−0.005	−0.003	−0.038	−0.016	0.111	0.092	0.035	0.017
		30	0.009	0.006	−0.037	−0.015	0.099	0.112	0.037	0.018
	MAR.nl	5	−0.008	−0.009	−0.037	−0.015	0.096	0.090	0.035	0.017
		15	−0.003	−0.001	−0.037	−0.015	0.111	0.084	0.037	0.018
		30	0.005	0.010	−0.039	−0.013	0.118	0.092	0.040	0.019
400	MCAR	5	−0.008	−0.009	−0.019	−0.008	0.040	0.037	0.015	0.006
		15	−0.011	−0.012	−0.019	−0.007	0.050	0.042	0.015	0.006
		30	−0.010	−0.010	−0.017	−0.006	0.065	0.048	0.015	0.006
	MAR.lin	5	−0.009	−0.009	−0.019	−0.008	0.037	0.032	0.015	0.006
		15	−0.009	−0.009	−0.018	−0.007	0.046	0.039	0.015	0.006
		30	−0.005	−0.006	−0.019	−0.007	0.055	0.049	0.016	0.006
	MAR.nl	5	−0.008	−0.008	−0.019	−0.008	0.037	0.030	0.015	0.006
		15	−0.008	−0.008	−0.019	−0.007	0.038	0.033	0.015	0.006
		30	−0.012	−0.009	−0.020	−0.008	0.062	0.051	0.017	0.007
600	MCAR	5	−0.007	−0.006	−0.010	−0.003	0.030	0.023	0.009	0.003
		15	−0.007	−0.006	−0.010	−0.003	0.032	0.025	0.009	0.003
		30	−0.008	−0.007	−0.010	−0.003	0.047	0.036	0.010	0.004
	MAR.lin	5	−0.007	−0.006	−0.010	−0.002	0.030	0.023	0.009	0.003
		15	−0.007	−0.007	−0.009	−0.002	0.032	0.025	0.008	0.002
		30	−0.008	−0.007	−0.009	−0.002	0.041	0.036	0.009	0.003
	MAR.nl	5	−0.008	−0.007	−0.011	−0.003	0.030	0.023	0.009	0.003
		15	−0.006	−0.006	−0.010	−0.002	0.033	0.026	0.009	0.003
		30	−0.008	−0.006	−0.010	−0.002	0.044	0.036	0.009	0.002

*Shaded cells contain bias values that are >0.05 in absolute value*.

If the performance of the FIGLS method were being evaluated on an absolute metric, the observed bias values would be deemed satisfactory. However, relative to TSML, the FIGLS method does show considerably more bias, consistently across most study conditions. This pattern persists even at the largest sample sizes. These results suggest that while both methods are unbiased asymptotically, in finite samples the simpler and more stable TSML method performs better, though these differences are more of theoretical than of practical interest.

### Efficiency of parameter estimates

Average empirical standard deviations of factor loadings and latent regression coefficient estimates are shown in Table 4. The winning method is bolded in each condition (each pair of FIGLS and TSML columns). In general, efficiency estimates are very similar for the two methods, and they are often identical at the largest studied sample size. However, it is clear that FIGLS's theoretical efficiency advantage does not as a general rule translate into an empirical efficiency advantage, at least in the conditions and sample sizes studied. At *N* = 600, FIGLS does have slightly smaller empirical standard deviations for factor loadings estimates in many conditions, but TSML has slightly smaller empirical standard deviations for latent regression coefficients. In smaller sample sizes, TSML tends to have smaller empirical standard deviations on average. The differences are again more of theoretical than of practical interest. Table 5 gives the RMSEs. The pattern of results here is very similar to that for bias (Table 3), which is unsurprising given that RMSEs are a joint measure of bias and efficiency, and the two methods did not differ very much on efficiency (Table 4). As with bias, TSML has smaller RMSEs than does FIGLS in most conditions, but both methods have low RMSEs.

**Table 4:** **Average efficiency estimates (empirical standard errors) for factor loadings and latent regression coefficients across all study conditions**.

***N***	**Missing**	**%**	**Factor loadings**				**Latent regression coefficients**			
	**mech**	**Miss**	**Model 1**		**Model 2**		**Model 1**		**Model 2**	
			**FIGLS**	**TSML**	**FIGLS**	**TSML**	**FIGLS**	**TSML**	**FIGLS**	**TSML**
200	MCAR	5	**0.282**	0.295	0.228	**0.227**	0.499	**0.439**	0.167	**0.156**
		15	0.315	0.315	0.237	**0.236**	0.615	**0.564**	0.181	**0.167**
		30	**0.374**	0.383	**0.254**	0.255	0.651	**0.650**	0.217	**0.185**
	MAR.lin	5	0.287	**0.282**	0.229	**0.228**	0.550	**0.481**	0.165	**0.156**
		15	0.307	**0.303**	0.237	**0.236**	0.588	**0.543**	0.171	**0.163**
		30	0.365	**0.359**	**0.256**	0.257	**0.554**	0.659	0.183	**0.178**
	MAR.nl	5	0.288	0.288	0.229	**0.228**	**0.492**	0.531	0.163	**0.156**
		15	**0.315**	0.319	0.240	**0.238**	0.614	**0.504**	0.177	**0.164**
		30	0.382	**0.370**	0.262	**0.261**	0.643	**0.624**	0.196	**0.183**
400	MCAR	5	0.198	**0.189**	0.158	0.158	**0.258**	0.318	0.103	**0.101**
		15	0.201	**0.200**	0.163	0.163	**0.271**	0.273	0.108	**0.106**
		30	0.230	**0.226**	0.173	0.173	0.394	**0.328**	0.110	**0.109**
	MAR.lin	5	0.189	**0.188**	0.158	0.158	**0.240**	0.260	0.104	**0.102**
		15	0.199	0.199	0.163	0.163	**0.325**	0.337	0.108	**0.106**
		30	**0.225**	0.228	0.175	0.175	**0.345**	0.368	0.113	**0.111**
	MAR.nl	5	0.189	0.189	0.159	0.159	**0.251**	0.256	0.104	**0.102**
		15	**0.201**	0.203	0.165	0.165	**0.248**	0.270	0.107	**0.105**
		30	**0.226**	0.227	0.178	0.178	0.372	**0.368**	0.115	**0.112**
600	MCAR	5	0.152	0.152	0.130	0.130	0.193	**0.192**	0.085	**0.085**
		15	**0.158**	0.159	0.133	0.133	0.202	**0.201**	0.086	**0.086**
		30	0.178	0.178	0.143	0.143	0.302	**0.258**	0.094	**0.093**
	MAR.lin	5	0.152	0.152	0.130	0.130	0.191	**0.187**	0.085	**0.084**
		15	**0.159**	0.160	0.134	0.134	0.199	**0.198**	0.088	**0.087**
		30	**0.178**	0.179	**0.143**	0.144	**0.233**	0.262	0.093	**0.092**
	MAR.nl	5	0.152	0.152	0.130	0.130	0.193	**0.190**	0.085	**0.085**
		15	0.161	0.161	0.135	0.135	0.206	**0.204**	0.088	**0.087**
		30	**0.181**	0.183	0.146	0.146	**0.274**	0.283	0.095	**0.094**

*The winning method is bolded in each condition*.

**Table 5:** **Average root mean square error estimates for factor loadings and latent regression coefficients across all study conditions**.

***N***	**Missing**	**%**	**Factor loadings**				**Latent regression coefficients**			
	**mech**	**Miss**	**Model 1**		**Model 2**		**Model 1**		**Model 2**	
			**FIGLS**	**TSML**	**FIGLS**	**TSML**	**FIGLS**	**TSML**	**FIGLS**	**TSML**
200	MCAR	5	0.011	**0.010**	0.037	**0.015**	0.093	**0.071**	0.035	**0.017**
		15	0.010	0.010	0.038	**0.016**	0.114	**0.085**	0.038	**0.019**
		30	**0.010**	0.016	0.041	**0.020**	0.132	**0.104**	0.046	**0.025**
	MAR.lin	5	0.013	**0.011**	0.037	**0.015**	0.105	**0.082**	0.034	**0.017**
		15	**0.010**	0.011	0.038	**0.016**	0.111	**0.092**	0.035	**0.017**
		30	**0.011**	0.012	0.037	**0.015**	**0.099**	0.112	0.037	**0.018**
	MAR.nl	5	0.012	0.012	0.037	**0.015**	0.096	**0.090**	0.035	**0.017**
		15	**0.010**	0.011	0.037	**0.015**	0.111	**0.084**	0.037	**0.018**
		30	**0.014**	0.020	0.039	**0.013**	0.118	**0.092**	0.040	**0.019**
400	MCAR	5	0.011	0.011	0.019	**0.008**	0.040	**0.037**	0.015	**0.006**
		15	0.014	**0.013**	0.019	**0.007**	0.050	**0.042**	0.015	**0.006**
		30	0.015	**0.014**	0.017	**0.006**	0.065	**0.048**	0.015	**0.006**
	MAR.lin	5	**0.010**	0.011	0.019	**0.008**	0.037	**0.032**	0.015	**0.006**
		15	0.010	0.010	0.018	**0.007**	0.046	**0.039**	0.015	**0.006**
		30	0.009	0.009	0.019	**0.007**	0.055	**0.049**	0.016	**0.006**
	MAR.nl	5	**0.009**	0.010	0.018	**0.007**	0.037	**0.030**	0.015	**0.006**
		15	0.010	0.010	0.019	**0.007**	0.038	**0.033**	0.015	**0.006**
		30	0.013	**0.011**	0.020	**0.008**	0.062	**0.051**	0.017	**0.007**
600	MCAR	5	0.009	**0.007**	0.010	**0.005**	0.030	**0.023**	0.009	**0.003**
		15	0.009	**0.008**	0.010	**0.005**	0.032	**0.025**	0.009	**0.003**
		30	0.010	**0.009**	0.010	**0.006**	0.047	**0.036**	0.010	**0.004**
	MAR.lin	5	0.008	**0.007**	0.010	**0.005**	0.030	**0.023**	0.009	**0.003**
		15	0.009	**0.008**	0.009	**0.006**	0.032	**0.025**	0.008	**0.004**
		30	0.010	**0.009**	0.009	**0.005**	0.041	**0.036**	0.009	**0.003**
	MAR.nl	5	0.009	**0.008**	0.011	**0.005**	0.030	**0.023**	0.009	**0.003**
		15	0.008	**0.007**	0.010	**0.006**	0.033	**0.026**	0.009	**0.004**
		30	0.009	**0.007**	0.010	**0.006**	0.044	**0.036**	0.009	**0.006**

*The winning method is bolded in each condition*.

### Coverage of 95% confidence intervals

Coverage is a joint measure of bias and the quality of the estimated (rather than empirical) standard errors (that is, how close these standard errors come to estimating the actual observed empirical efficiency). Coverage results are shown in Table 6. Coverage rates <93% are bolded, whereas coverage rates >97% are italicized. With factor loadings, FIGLS has optimal coverage in all study conditions. TSML does mostly well, but exhibits lower than optimal coverage in some conditions (never dropping below 93%, however), particularly when the sample size is small or the proportion of missing data is large. Regarding latent regression coefficients, TSML does not do well in Model 1, exhibiting coverage as low as 88% for the most difficult intersection of conditions. This behavior improves with sample size but coverage for TSML is frequently below optimal even at *N* = 600. On the other hand, FIGLS has near optimal coverage in almost all conditions. At *N* = 200, coverage for FIGLS tends to be a bit high, exceeding 97% in a few conditions.

**Table 6:** **Average coverage for factor loadings and latent regression coefficients across all study conditions**.

***N***	**Missing**	**%**	**Factor loadings**				**Latent regression coefficients**			
	**mech**	**Miss**	**Model 1**		**Model 2**		**Model 1**		**Model 2**	
			**FIGLS**	**TSML**	**FIGLS**	**TSML**	**FIGLS**	**TSML**	**FIGLS**	**TSML**
200	MCAR	5	95.9	94.4	94.6	94.0	94.4	**90.9**	*97.5*	95.8
		15	96.1	94.3	94.6	94.0	93.2	**89.7**	97.0	95.2
		30	95.8	93.8	94.2	93.6	**92.7**	**88.6**	96.9	95.4
	MAR.lin	5	95.7	94.4	94.7	94.2	94.5	**91.1**	*97.5*	96.3
		15	95.6	94.0	94.3	93.9	93.0	**90.5**	*97.2*	95.6
		30	95.3	93.2	94.3	93.4	93.1	**89.9**	96.8	95.3
	MAR.nl	5	95.8	94.3	94.6	94.0	93.9	**90.9**	*97.5*	96.2
		15	95.8	94.4	94.2	93.5	94.0	**90.8**	97.0	95.3
		30	95.9	93.7	94.1	93.5	93.4	**88.5**	96.4	94.8
400	MCAR	5	95.3	94.3	94.8	94.7	94.7	**92.8**	96.6	95.9
		15	94.9	93.8	94.5	94.6	94.5	**92.3**	96.4	95.7
		30	95.0	93.8	94.8	94.7	94.9	**92.2**	96.4	95.8
	MAR.lin	5	95.0	94.1	94.6	94.6	95.4	93.8	96.6	95.8
		15	95.2	94.2	94.7	94.6	94.9	93.1	96.5	95.4
		30	95.2	94.2	94.4	94.2	94.0	**91.7**	96.1	95.2
	MAR.nl	5	95.2	94.3	94.5	94.4	95.2	93.5	96.2	95.6
		15	95.3	94.1	94.7	94.7	94.6	**92.9**	96.7	95.6
		30	95.3	94.2	94.4	94.1	94.8	**91.9**	96.2	95.4
600	MCAR	5	94.9	94.4	94.7	94.5	94.4	93.1	95.5	95.0
		15	95.0	94.5	94.9	94.7	94.2	**92.7**	95.0	94.3
		30	94.6	94.1	94.5	93.9	93.7	**92.3**	94.7	93.8
	MAR.lin	5	94.7	94.2	94.7	94.4	94.5	93.4	95.3	94.5
		15	94.9	94.4	94.7	94.4	94.2	93.5	95.2	94.3
		30	94.8	94.3	94.5	94.3	94.0	**92.5**	95.3	94.6
	MAR.nl	5	94.6	94.2	94.8	94.5	94.3	93.1	95.2	94.8
		15	94.7	94.2	94.6	94.4	93.8	93.1	95.4	94.5
		30	94.8	94.0	94.5	94.0	94.0	**92.6**	94.2	93.8

*Coverage rates <93% are bolded. Coverage rates >97% are italicized*.

### Type i error rates

Rejection rates of the chi-square tests of fit are given in Table 7. Values below 4% and above 6% are bolded. While there are quite a few conditions where the rates deviate from 5% by more than 1 percentage point, this deviation is never very strong. Rejection rates never exceed 6.7% in any condition. The lowest observed value is 2.3%. In general, under-rejection was most common under Model 1 with *N* = 200. Most importantly for the present paper, there is not much difference in rejection rates across the two methods. The normal theory residual-based chi-square associated with the TSML estimator and the usual minimum fit function chi-square associated with the FIGLS estimator appear to produce highly similar rejection rates. Of course, both statistics are asymptotically chi-square distributed with normal data, but in even in small samples, the differences are minor.

**Table 7:** **Type I error rates across all study conditions**.

***N***	**Missing**	**%**	**Model 1**		**Model 2**	
	**mech**	**Miss**	**FIGLS**	**TSML**	**FIGLS**	**TSML**
200	MCAR	5	**3.0**	**3.3**	5.7	5.7
		15	**3.9**	4.4	4.8	5.0
		30	**6.5**	**6.9**	5.1	5.2
	MAR.lin	5	**3.0**	**3.1**	4.6	5.0
		15	**3.0**	**3.8**	**6.1**	**6.2**
		30	5.4	**6.1**	**6.5**	**6.6**
	MAR.nl	5	**2.3**	**3.0**	4.7	4.6
		15	**3.2**	**3.3**	4.7	4.7
		30	4.2	4.3	5.2	5.3
400	MCAR	5	4.8	4.8	**3.1**	**3.1**
		15	4.8	5.0	4.9	4.9
		30	**6.1**	**6.2**	4.3	4.3
	MAR.lin	5	4.1	4.0	**3.6**	**3.6**
		15	4.9	5.0	4.3	4.3
		30	**6.5**	**6.7**	**3.7**	**3.8**
	MAR.nl	5	4.7	4.6	**3.4**	**3.4**
		15	5.4	5.4	4.8	4.9
		30	5.5	5.5	5.0	5.0
600	MCAR	5	5.5	5.5	4.2	4.1
		15	5.6	5.6	4.6	4.6
		30	5.0	5.0	4.9	4.9
	MAR.lin	5	5.9	6.0	**3.7**	**3.7**
		15	5.4	5.4	4.4	4.3
		30	5.1	5.2	4.7	4.7
	MAR.nl	5	5.4	5.5	4.3	4.3
		15	**6.5**	**6.5**	4.8	4.8
		30	6.0	**6.1**	4.9	4.9

*Rejection rates below 4% and above 6% are bolded*.

## Discussion

This article proposed a new missing data estimator for incomplete normally distributed data, “full information” generalized least squares (FIGLS). The new estimator is the generalization of the GLS estimator to incomplete data. With complete data, the GLS estimator is not used often, because it tends to be outperformed by ML (e.g., Olsson et al., 2000). However, an extension to incomplete data has not been proposed or studied before. Importantly, the FIGLS estimator is further extended to be applicable to item-level missing data. Item-level missing data arise in many contexts. In the context of regression or path analysis, variables in the model are often scale scores composed of individual items. In the context of SEM, latent variable models are often built for parcels (sums of indicators) and not for raw indicators. Parcels are particularly useful when the sample size is small and when the measurement model is not of direct interest (Little et al., 2013). In both these examples, item-level missing data are quite likely to occur. Common *ad-hoc* solutions such as computing composite scores based on all available items or treating the composite score as missing if any of the items are missing are unsatisfactory, as they lose efficiency at best and create bias at worst.

In contrast, the newly proposed FIGLS estimator yields consistent parameter estimates under MAR and is asymptotically efficient. The comparison method used in the simulation presented here, two-stage ML (TSML; Savalei and Rhemtulla, 2017), is also consistent under MAR, but is not asymptotically efficient, though its efficiency loss is very small. TSML is essentially the analytic equivalent of item-level multiple imputation. It is worth repeating that the most popular analytic method for missing data treatment, FIML, is not possible for item-level missing data without invoking an item-level model that would be estimated first—as in most cases this model is not of direct interest to the researcher, this method is not desirable.

This article also presented the results of a simulation study comparing FIGLS and TSML with item-level missing data in the context of an SEM with parcels. These results show that the two methods perform very similarly. From a practical standpoint, either method can be successfully used with item-level missing data, and would represent a vast improvement over *ad-hoc* approaches. Bias in parameter estimates was in general negligible for factor loadings but was considerable for latent regression coefficients at the smallest sample size; however, it diminished quickly with increasing sample size. Coverage was worse for latent regression coefficients than for factor loadings, and in general required a larger sample size. Rejection rates were acceptable and sufficiently close to nominal in most study conditions.

The differences between the methods were usually minor from a practical standpoint. TSML exhibited a much smaller bias than FIGLS in many conditions, but while this difference is theoretically interesting, it was of little practical significance. Efficiency estimates for FIGLS and TSML were quite similar. FIGLS had optimal coverage for both factor loadings and latent regression coefficients in most conditions, while TSML had some suboptimal coverage in some conditions, particularly for latent regression coefficients. The methods did not differ much in terms of the chi-square statistic rejection rates.

This study represents a first preliminary investigation of the GLS approach to missing data. Further study of FIGLS is encouraged. TSML is also a relatively new method for item-level missing data that requires further study. We hope that both of these methods will soon be automated in popular software. We share sample code that is implemented in *R* (and heavily relies on the *lavaan* package) on the Open Science Framework.

## Author contributions

VS developed the estimator and wrote the first draft of the manuscript. VS and MR designed and carried out the simulation study. MR assisted with revisions of the manuscript.

## Funding

This research was supported by Natural Sciences and Engineering Research Council of Canada Grant RGPIN-2015-05251 to VS and Marie Curie Career Integration Grant PCIG14-GA-2013-631145 to MR.

### Conflict of interest statement

The authors declare that the research was conducted in the absence of any commercial or financial relationships that could be construed as a potential conflict of interest.
